# Massively parallel sequencing of formalin-fixed paraffin-embedded tissues

**DOI:** 10.1186/1753-6561-6-S6-P40

**Published:** 2012-10-01

**Authors:** Rachel L Erlich, Emanuele Palescandolo, Robert T Jones, Ashwini Sunkavalli, Alina Raza, Matthew Ducar, Charles Hatton, Christina Go, Christine Roden, Megan Hanna, Adri Mills, Ravali Adusumilli, Prateek Kumar, Priscilla K Brastianos, Matthew L Meyerson, William C Hahn, Laura E MacConaill, Paul Van Hummelen

**Affiliations:** 1 Dana-Farber Cancer Institute, Boston, Massachusetts 02115, USA

## 

Formalin fixation followed by paraffin embedment (FFPE) is the most common method of preserving resected tissues. FFPEs can be easily stored, retrieved and processed for further analysis when compared to the logistical complications of processing fresh frozen (FF) material. Conversely, they may not be ideal for sequencing because of the nucleic acid fragmentation and artifacts introduced by fixation. Here we report our efforts in performing next-generation sequencing on more than 70 specimens comprising >40 FFPE samples, from low to high quality, and compared the performance of FFPE, FF and commercially available cell lines.

